# Optimization of Multiple and Multipurpose Reservoir System Operations by Using Matrix Structure (Case Study: Karun and Dez Reservoir Dams)

**DOI:** 10.1371/journal.pone.0156276

**Published:** 2016-06-01

**Authors:** Mohammad Heydari, Faridah Othman, Mahmood Taghieh

**Affiliations:** 1 Department of Civil Engineering, Faculty of Engineering, University Malaya, Kuala Lumpur, Malaysia; 2 Department of Civil Engineering, Faculty of Engineering, University Malaya, Kuala Lumpur, Malaysia; 3 Department of Industrial Engineering, Faculty of Engineering, Amirkabir University, Tehran, Iran; Nankai University, CHINA

## Abstract

Optimal operation of water resources in multiple and multipurpose reservoirs is very complicated. This is because of the number of dams, each dam’s location (Series and parallel), conflict in objectives and the stochastic nature of the inflow of water in the system. In this paper, performance optimization of the system of Karun and Dez reservoir dams have been studied and investigated with the purposes of hydroelectric energy generation and providing water demand in 6 dams. On the Karun River, 5 dams have been built in the series arrangements, and the Dez dam has been built parallel to those 5 dams. One of the main achievements in this research is the implementation of the structure of production of hydroelectric energy as a function of matrix in MATLAB software. The results show that the role of objective function structure for generating hydroelectric energy in weighting method algorithm is more important than water supply. Nonetheless by implementing ε- constraint method algorithm, we can both increase hydroelectric power generation and supply around 85% of agricultural and industrial demands.

## Introduction

Large dams are usually built for different purposes such as urban water supply, industrial, agricultural, power generation, flood control, environmental objectives, navigation etc. Recently, much research has been done to achieve certain objectives in optimal reservoir operation; such as optimizing hydroelectric power [1, 2], flood control [3], irrigation [4, 5], and environmental [6–8]. The main research methodologies are about achieving the optimum level of release and optimal storage volume by considering the changes in inflow and needs [9].

In the past few years, researchers used different methods to achieve the mentioned objectives like Linear Programming (LP) or Evolutionary Algorithms (EA) such as Genetic Algorithms (GA). Comprehensive reviews of these techniques have been written several years ago, for instance, Yeh [10], Wurbs [11] and Labadie [12]. However, due to the physical and operational characteristics, a unique algorithm cannot be selected as the best standard technique [10].

Linear programming is widely used in the modeling of Multi-Reservoir Operation Planning (MROP) problems [11]. This is mostly because most scientific problems are presented in a linear programming frame. Conversion of problem to matrix (to solve it by matrix software’s) and having a set of software like LINGO or GAMS and also spreadsheets like Excel to solve such problems with the ability of considering thousands of variables are prominent advantages of linear programming.

Since it is possible that objective functions and some of the constraints are nonlinear, fragmentation linear techniques have been used such as approximation by Taylor series to linearize the objective functions and constraints [13]. Fragmentation linear programming is dependent on the capabilities of the separation of objective function and limitations, if the line of succession does not require this assumption. Studies done have shown that the programming problems regarding the performance of reservoir dams is a random non-linear programming problem, especially if the objective function is the production function of hydroelectric energy [14, 15].

The main factor in determining the optimal operation system of a dam—including single dam and multiple dam—is the nonlinear relationship between hydropower energy production, the amount of water released from turbines under uncertainty conditions of input flows and the amount of demands for electrical energy [14, 16]. The optimization model for planning operating systems of multiple dams should reflect the exchange between the benefits obtained from the storage and saving of the water and the benefits obtained from releasing the water. On the other hand, there is an exchange between the benefits of storing water in high-level and the loss resulting from the overflow of water. This study’s aim is to create and develop a general flexible model which includes the structure and main features of the problem as much as possible.

## Materials and Methods

### Case Study

**Fig 1:**
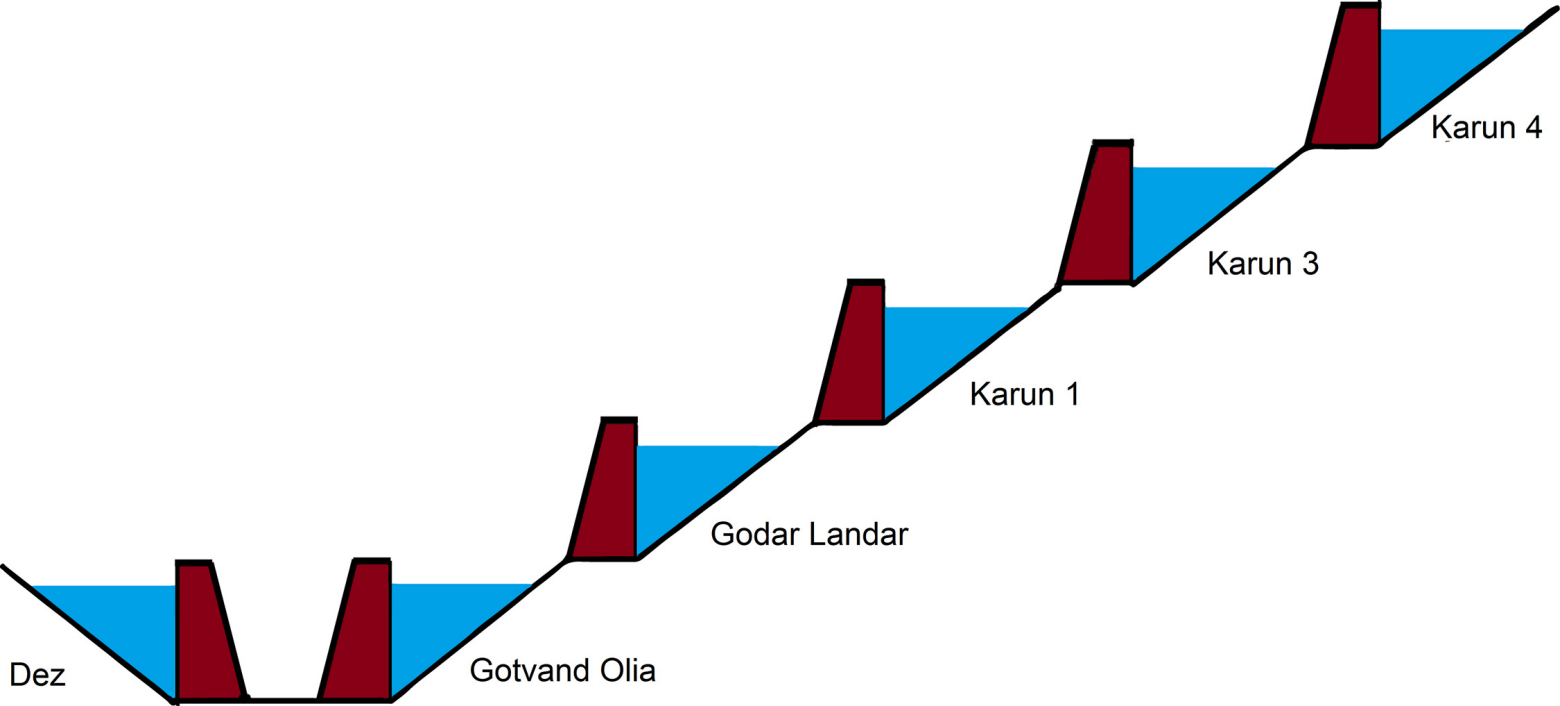
Dez and Karun rivers system details.

In this study, a series of reservoir dams in Karun and Dez were investigated. The Dez and Karun river basins are located in south-western Iran, which includes more than a fifth of the country's watershed basins [17]. The total catchment area of these two rivers is approximately 45,000 square kilometers [18]. The system consisting of 6 reservoir dams are either built or are under construction (Fig 1). Five of these successive dams have been constructed on the Karun River and a dam has been constructed on the Dez River which is parallel to the Karun Dam series. The two river confluence in the northern city of Ahvaz in Bandghir area and form a large Karun River which leads to the Persian Gulf. In this study, only the current dam system and the dams under construction are modeled. The total area of agricultural land is about 250,000 square kilometers in this location. The main purposes of the construction of a series of dams can be summarized as: the production of hydroelectric energy, to meet agricultural and industrial consumer needs in the area and to control seasonal flooding. (No specific permissions were required for these locations/activities)

### Problem Description and Formulation (Constraints and Objective Functions)

The structure of the model is designed in such a way that it has the ability to be planned for long and short terms. However, the model cannot contain all extended details. The extensive details may be essential to show all physical limitations of a system of reservoir dam in the real world.

The objective functions considered in this study include:

1. Minimizing allocation deviations of water demand for agricultural and industrial purposes
2. Maximizing the production of hydroelectric energy

Variables that determine the structure and state of the system include: the volume of water stored in the dam, amount of water passing through the turbines, the overflow of water coming out of the spillway and the percentage of supply of demanded water in each period. The relationship between the independent variables and the input flow amount is created by the flow equations in each period. Decision variables of the problem are the amount of water released from the turbine canals and the amount of output water from sub-channels of each of the dams in each planning period.

Generally, the following equation can be considered as the flow equation in a system of reservoir dams in a planning period:

S(t)=S(t−1)−[Rpower(t)+Spill(t)]+I(t) (1)

Where t is defined as period of time. S(t) is defined as the volume of water stored in the current period. S(t -1) is defined as the volume of water stored in the previous period. Rpower(t) is defined as the volume of water released through the turbines during t period. Spill(t) is defined as the volume of water overflow during t period and I(t) is defined as the total volume of natural flow into dams during t period.

In this equation, the effect of variables, such as the rate of evaporation and the amount of water absorbed by the earth is not considered. In a system where dams are located in series or parallel, output water flows from the turbines and spillway along the natural flows of the middle basin pour into other dams after allocating water for agriculture and industry needs in the middle basin.

In addition to the flow equations, there are some relationships such as flow non-equations that constitute the decision variables in a reservoir dam. Because the volume of water allocated for agriculture and industry cannot be more than the total volume of the output stream from the dam upstream and natural streams entering into the dam in each dam, the following non-equation can be considered among the decision variables in the system:

−[Rpower(t)+Spill(t)]+dem(t).α(t)≤B(t) (2)

Where t is the period of time. Rpower(t) is the volume of water passing through the turbines during t period. Spill(t) is the volume of overflow water during t period. *α*(t) is the percentage of water allocated to the middle basin during t period. dem(t) is the rate of demand in the middle basin during t period and B(t) is the volume of water flow between the path of the middle basin during t period.

In general, there are two types of decision variables in a mathematical model. The first kind is a variable that has not imposed any certain limitation and the other is a kind that can only be accepted in certain amounts. The first type is called an infinite variable and the second type is called a bounded variable. This bound may appear in the form of an upper bound or lower bound or both in the model.

Eqs (1) and non-equation (2) have upper and lower bounds because of the physical structure of each dam and the variables: volume of water stored in each period, volume of water passing through the turbines and the volume of spillways which are provided as follows:

$$S_{min}\leq S(t)\leq S_{max}$$ (3)

$$Rpower_{min}\leq Rpower(t)\leq Rpower_{max}$$ (4)

$$Spill_{min}\leq Spill(t)\leq Spill_{max}$$ (5)

Where Smax is considered as the maximum capacity of the reservoir, Smin is considered as the minimum capacity of the reservoir, Rpowermax is considered as the maximum capacity of the tunnels which direct water into the powerhouse, Rpowermin is considered as the minimum discharge of the turbines designed, Spillmax is considered as the maximum capacity of the dam spillway and Spillmin is considered as zero. In addition to the above constraints in the set of flow equations, the percentage variable of the supply demand rate in each area has been defined for different periods in order to supply water in the middle area. Allocation of water for agricultural and industry usage is determined in each period by multiplying this variable by the demand rate in each area. As a result, the upper and lower bounds for this variable are defined as follows:

$$\alpha_{min}\leq \alpha (t)\leq 1$$ (6)

In order to meet the demand for water in the middle basin which consists mainly of industrial, agricultural and drinking usage; the defined objective function must maximize the allocation of water for the purposes mentioned. Actually, the total allocated diversion demands should be minimized, because the amount of allocated water is less than or equal to the demand rate. This relationship can be presented as follows:

Min∑(Demand−Allocation) (7)

According to the definition of variable α which is the percentage supply of water demanded and with attention to planning courses, Eq (7) can be offered as follows:

$$Min\sum_{t=1}^{T}\left(dem(t)-dem(t).\alpha (t)\right)$$ (8)

$$Max\sum_{t=1}^{T}\left(dem(t).\alpha (t)-dem(t)\right)$$ (9)

The hydroelectric energy production function is a nonlinear function of the average water height stored behind a dam and the amount of release through the turbine in a reservoir dam [6]. The nonlinear relationship can be presented as follows:

$$Power\left(t\right)=F(\bar{H}\left(t\right).Rpower\left(t\right))=C.e.\bar{H}\left(t\right).Rpower\left(t\right)$$ (10)

Where Power(t) is defined as the energy produced per Mega Watt Hour (MWh) during t period, H(t) is defined as the average height of the water stored behind the dam during t period, Rpower(t) is defined as the volume of water passing through the turbine during t period, e is defined as the efficiency of the powerhouse and C is defined as the energy conversion coefficient. By increasing the amount of water being released, the water height will increase at the bottom of the dam (coastal) which causes the generated energy to be lost. As a result, the effective height is considered instead of the water height behind the dam therefore, Eq (10) is defined as follows:

$$Power\left(t\right)=F(\bar{H}\left(t\right).Rpower\left(t\right))=C.e.{\bar{H}}_{{}_{e}}\left(t\right).Rpower\left(t\right)$$ (11)

$$Power\left(t\right)=C.e.(H_{total}\left(t\right)-H_{tail}(t)).Rpower\left(t\right)$$ (12)

The water height behind the dam is a function of the reservoir’s storage, the height of water in the tailwater is also a function of the releasing rate. These relations can be considered as follows:

$$H_{total}\left(S\right)=a+b.S\;,\;S\geq S_{min}\;;a,b>0$$ (13)

$$H_{tail}\left(Rpower\right)=c+d.Rpower\;,\;Rpower\geq Rpower_{min}\;;c,d>0$$ (14)

By substituting Eqs (13) and (14) into Eq (12) we get:

$$Power\left(t\right)=C.e.(\left(a-c\right).Rpower\left(t\right)+b.S\left(t\right).Rpower\left(t\right)-d.\left(Rpower{\left(t\right)}^{2}\right)$$ (15)

### Performance optimization model of a series of reservoir dams; Karun and Dez

According to the Eqs (1) to (15), the Performance optimization Model of the Series of Reservoir Dams in Karun and Dez will be as follows:

$$MaxF_{1}=\sum_{i=1}^{6}\sum_{t=1}^{T}C_{i}\text{.}e_{i}\text{.}[(a_{i}-c_{i}).Rpower_{i}(t)+b_{i}.S_{i}(t).Rpower_{i}(t)-d_{i}.(Rpower{\left(t\right)}^{2})]$$ (16)

$$MaxF_{2}=\sum_{j=1}^{7}\sum_{t=1}^{T}\left(dem_{j}(t).\alpha_{j}(t)-dem_{j}(t)\right)$$ (17)

S.t:

$$S_{1}\left(t\right)-S_{1}\left(t-1\right)+\left[Rpower_{1}\left(t\right)+Spill_{1}\left(t\right)\right]=I_{1}\left(t\right)$$ (18)

$$S_{2}\left(t\right)-S_{2}\left(t-1\right)+\left[Rpower_{2}\left(t\right)+Spill_{2}\left(t\right)\right]-\left[Rpower_{1}\left(t\right)+Spill_{1}\left(t\right)\right]+dem_{1}\left(t\right).\alpha_{1}\left(t\right)=I_{2}\left(t\right)$$ (19)

$$S_{3}\left(t\right)-S_{3}\left(t-1\right)+\left[Rpower_{3}\left(t\right)+Spill_{3}\left(t\right)\right]-\left[Rpower_{2}\left(t\right)+Spill_{2}\left(t\right)\right]+dem_{2}\left(t\right).\alpha_{2}\left(t\right)=I_{3}\left(t\right)$$ (20)

$$S_{4}\left(t\right)-S_{4}\left(t-1\right)+\left[Rpower_{4}\left(t\right)+Spill_{4}\left(t\right)\right]-\left[Rpower_{3}\left(t\right)+Spill_{3}\left(t\right)\right]+dem_{3}\left(t\right).\alpha_{3}\left(t\right)=I_{4}\left(t\right)$$ (21)

$$S_{5}\left(t\right)-S_{5}\left(t-1\right)+\left[Rpower_{5}\left(t\right)+Spill_{5}\left(t\right)\right]-\left[Rpower_{4}\left(t\right)+Spill_{4}\left(t\right)\right]+dem_{4}\left(t\right).\alpha_{4}\left(t\right)=I_{5}\left(t\right)$$ (22)

$$S_{6}\left(t\right)-S_{6}\left(t-1\right)+\left[Rpower_{6}\left(t\right)+Spill_{6}\left(t\right)\right]=I_{6}\left(t\right)$$ (23)

$$\begin{array}{l} S_{7}\left(t\right)-\left[Rpower_{5}\left(t\right)+Spill_{5}\left(t\right)\right]-\left[Rpower_{6}\left(t\right)+Spill_{6}\left(t\right)\right]+\\ {}[dem_{5}\left(t\right).\alpha_{5}\left(t\right)+dem_{6}\left(t\right).\alpha_{6}\left(t\right)+dem_{7}\left(t\right).\alpha_{7}\left(t\right)]=I_{7}\left(t\right) \end{array}$$ (24)

$$-\left[Rpower_{1}\left(t\right)+Spill_{1}\left(t\right)\right]+dem_{1}\left(t\right).\alpha_{1}\left(t\right)\leq B_{1}\left(t\right)$$ (25)

$$-\left[Rpower_{2}\left(t\right)+Spill_{2}\left(t\right)\right]+dem_{2}\left(t\right).\alpha_{2}\left(t\right)\leq B_{2}\left(t\right)$$ (26)

$$-\left[Rpower_{3}\left(t\right)+Spill_{3}\left(t\right)\right]+dem_{3}\left(t\right).\alpha_{3}\left(t\right)\leq B_{3}\left(t\right)$$ (27)

$$-\left[Rpower_{4}\left(t\right)+Spill_{4}\left(t\right)\right]+dem_{4}\left(t\right).\alpha_{4}\left(t\right)\leq B_{4}\left(t\right)$$ (28)

$$-\left[Rpower_{5}\left(t\right)+Spill_{5}\left(t\right)\right]+dem_{5}\left(t\right).\alpha_{5}\left(t\right)\leq B_{5}\left(t\right)$$ (29)

$$-\left[Rpower_{6}\left(t\right)+Spill_{6}\left(t\right)\right]+dem_{6}\left(t\right).\alpha_{6}\left(t\right)\leq B_{6}\left(t\right)$$ (30)

$$\begin{array}{l} -\left[Rpower_{5}\left(t\right)+Spill_{5}\left(t\right)\right]+dem_{5}\left(t\right).\alpha_{5}\left(t\right)-\left[Rpower_{6}\left(t\right)+Spill_{6}\left(t\right)\right]\\ +dem_{6}\left(t\right).\alpha_{6}\left(t\right)+dem_{7}\left(t\right).\alpha_{7}\left(t\right)\leq B_{5}\left(t\right)+B_{6}\left(t\right)+B_{7}\left(t\right) \end{array}$$ (31)

$$S_{i}^{\text{min}}\leq S_{i}(t)\leq S_{i}^{\text{max}}$$ (32)

$$Rpower_{i}^{\text{min}}\leq Rpower_{i}(t)\leq Rpower_{i}^{\text{max}}$$ (33)

$$Spill_{i}^{\text{min}}\leq Spill_{i}(t)\leq Spill_{i}^{\text{max}}$$ (34)

$$R_{7}^{\text{min}}\leq R_{7}(t)$$ (35)

$$\alpha_{j}^{\text{min}}\leq \alpha_{i}(t)\leq 1$$ (36)

### Implementing a Matrix Structure of Optimization Model

In order to implement the matrix structure model of planned operation for the Karun and Dez dams, the matrix model is considered as a set of constraints and objective functions based on the proposed structure model as following:

$$Max\;F_{1}=C_{1}{}^{T}.x\;+.x^{T}.H_{1}.x$$ (37)

$$Max\;F_{2}=C_{2}{}^{T}.x$$ (38)

*S*.*t*.:

Aeq.x=beq (39)

Aineq.x≤bineq (40)

$$L_{b}\leq x\leq U_{b}$$ (41)

Where: x is a decision vector, C_1_ is the coefficient vector of the linear part of the function of energy production, H_1_ is a Hessian matrix of the non-linear production function, C_2_ is the coefficient vector of the demand for water supply, A_eq_ is the flow equations Matrix, b_eq_ is the Right vector of the flow equations, A_ineq_ is the non-equations Matrix, U_b_ is the opposite top of decision variables and L_b_ is the opposite bottom of decision variables.

Decision vector x including decision variables can be demonstrated as follows:

$$x=\left[\begin{array}{l} S\\ Rpower\\ Spill\\ \alpha \end{array}\right]$$ (42)

Now, due to the provided set of equations, it is possible to consider matrix Aeq as follows:

Aeq=[ASeq,ARpowereq,ASpilleq,Aalphaeq] (43)

The flow non-equations cannot be presented as a matrix:

Aineq=[ASineq,ARpowerineq,ASpillineq,Aalphaineq] (44)

The hydroelectric energy production objective function given in (15) has two separate linear and nonlinear parts. Based on the definition of decision vector x, the coefficient vector of linear part of the function is considered as follows:

$$C_{1}=\left[\begin{array}{l} {\left(zero\right)}_{6*1}\\ a_{1}-c_{1}\\ a_{2}-c_{2}\\ a_{3}-c_{3}\\ a_{4}-c_{4}\\ a_{5}-c_{5}\\ a_{6}-c_{6}\\ 0\\ {\left(zero\right)}_{7*1}\\ {\left(zero\right)}_{7*1} \end{array}\right]$$ (45)

The nonlinear function of hydroelectric power can also be presented as a Hessian matrix. The Hessian matrix structure of the objective function which is a block matrix can be considered in the following form:

$$\text{H}1=\left[\begin{array}{lllllllll} \; & \; & \; & b1 & 0 & 0 & 0 & \; & \;\\ \; & {\left(0\right)}_{6*6} & & 0 & \ddots & \vdots & 0 & {\left(0\right)}_{7*7} & {\left(0\right)}_{7*7}\\ \; & & \; & 0 & \; & b6 & 0 & \; & \;\\ b1 & 0 & 0 & d1 & 0 & 0 & 0 & \; & \;\\ 0 & \ddots & \vdots & 0 & \ddots & \vdots & 0 & {\left(0\right)}_{7*7} & {\left(0\right)}_{7*7}\\ 0 & 0 & b6 & 0 & 0 & d6 & 0 & \; & \;\\ 0 & 0 & 0 & 0 & 0 & 0 & 0 & \; & \;\\ \; & {\left(0\right)}_{7*6} & \; & \; & {\left(0\right)}_{7*7} & \; & \; & {\left(0\right)}_{7*7} & {\left(0\right)}_{7*7}\\ \; & {\left(0\right)}_{7*6} & \; & \; & {\left(0\right)}_{7*7} & \; & \; & {\left(0\right)}_{7*7} & {\left(0\right)}_{7*7} \end{array}\right]$$ (46)

(zero) _m_×_n_: Zero matrix contains m row and n column, i: Index of Dams: i = 1,2,3,4,5,6:ai,bi,ci,di constant and positive model coefficients, which are determined based on the physical structure of each dam.

The matrix structure of objective functions and constraints set of the optimization model of Karun-Dez system was implemented in software MATLAB. Due to the high dimensions of the problem like the number of variables, equations and non-equations in the constraints set, the non-linear structure of the supply of consumption needs area and hydroelectric energy production. Due to the structure of the set of constraints and objective functions, these matrices were implemented as sparse software. In addition to implementing these matrices, programs were created in the model based on the physical structure of the dam, its location and type of turbine were used to determine the coefficients of the objective function regarding hydroelectric energy production. This program adjusts matrix dimensions of the problem based on the planning period. It should be noted that the dimensions of the problem include 12,960 variables and 6,720 linear constraints by using data from a 40-year period.

The MATLAB software was used to optimize the model with the objective function of supplying water and hydroelectric energy generation. This software optimizes the large-scale linear programming and nonlinear problems by using interior points and utilizing the structure of the sparse matrix [19]. The algorithms that have been developed on the basis mentioned, have very high efficiency and flexibility. The results of the optimization matrix are discussed using multi-criteria planning techniques.

## Results and Discussion

### Weighting Method Algorithm

In this method, by assigning a non-negative weight to each objective, we try to optimize the total weighted function. In addition, in this method multi objective problems becomes single-objective problem.

$$Min\;w_{1}f_{1}+w_{2}f_{2}+\ldots +w_{n}f_{n}$$ (47)

S.t.g(x)≤0 (48)

h(x)=0 (49)

In the following is a two-objective programming problem model for Karun-Dez:

$$MaxF_{1}={\sum_{i=1}^{6}\sum_{t=1}^{T}Power}_{i}(t)$$ (50)

$$MaxF_{2}=\sum_{j=1}^{7}\sum_{t=1}^{T}\left(dem_{j}(t).\alpha_{j}(t)-dem_{j}(t)\right)$$ (51)

$$s.t.:F_{d}\leq 0$$ (52)

By using the weighted method, the optimization problem is converted into a single-objective programming problem:

$$MaxF=\lambda_{1}.F_{1}+\lambda_{2}.F_{2}$$ (53)

$$MaxF=\lambda_{1}.[{\sum_{i=1}^{6}\sum_{t=1}^{T}Power}_{i}(t)]+\lambda_{2}.[\sum_{j=1}^{7}\sum_{t=1}^{T}\left(dem_{j}(t).\alpha_{j}(t)-dem_{j}(t)\right)]$$ (54)

$$s.t.:F_{d}\leq 0$$ (55)

Then, based on the different λ_1_ and λ_2_ the optimal solution of problem (54) is determined. The optimal answers for this problem are the same answers as non dominated answers in the question (50 and 51).

**Fig 2:**
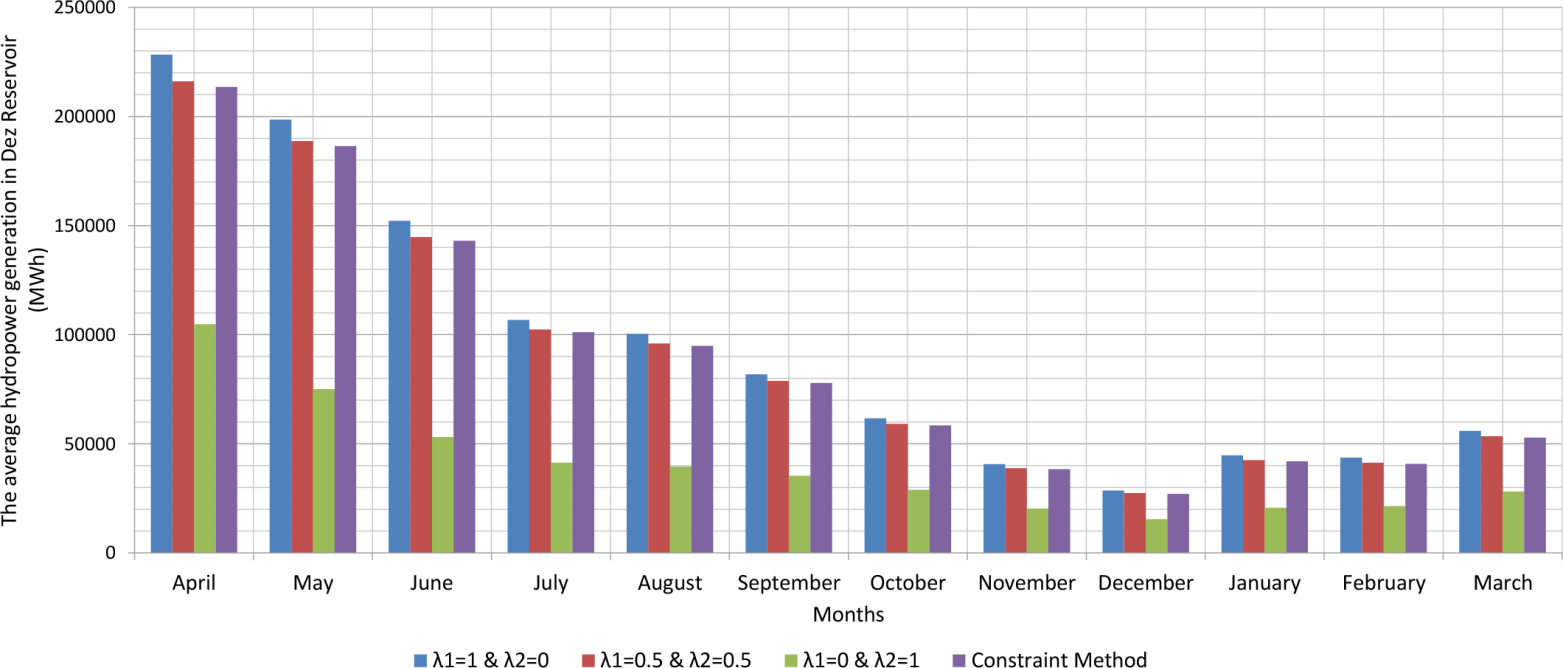
The average hydropower generation in Dez reservoir (MWh).

**Fig 3:**
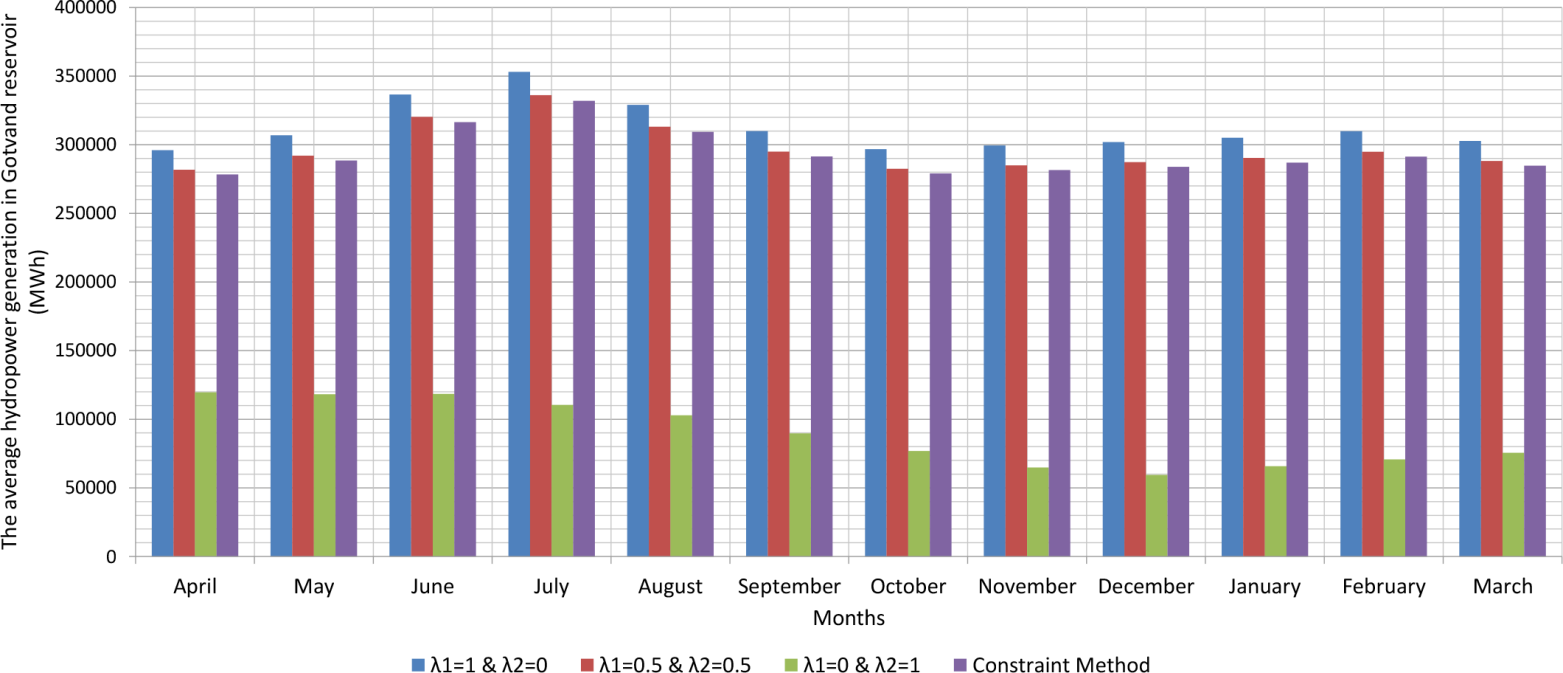
The average hydropower generation in Gotvand reservoir (MWh).

**Fig 4:**
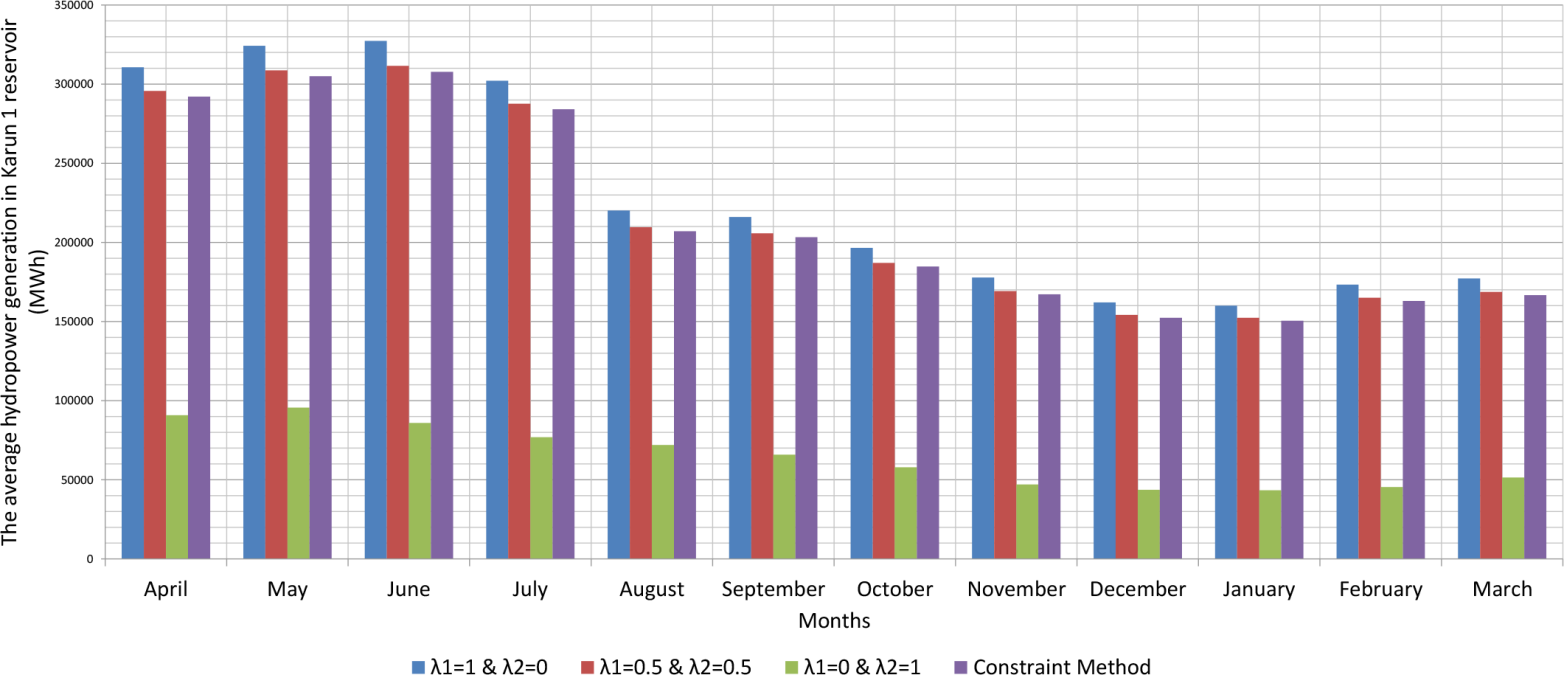
The average hydropower generation in Karun 1 reservoir (MWh).

**Fig 5:**
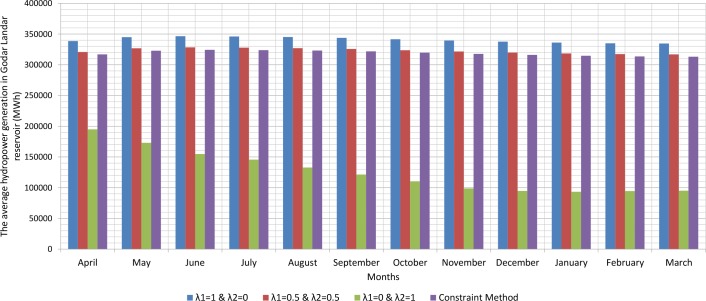
The average hydropower generation in Godar Landar reservoir (MWh).

**Fig 6:**
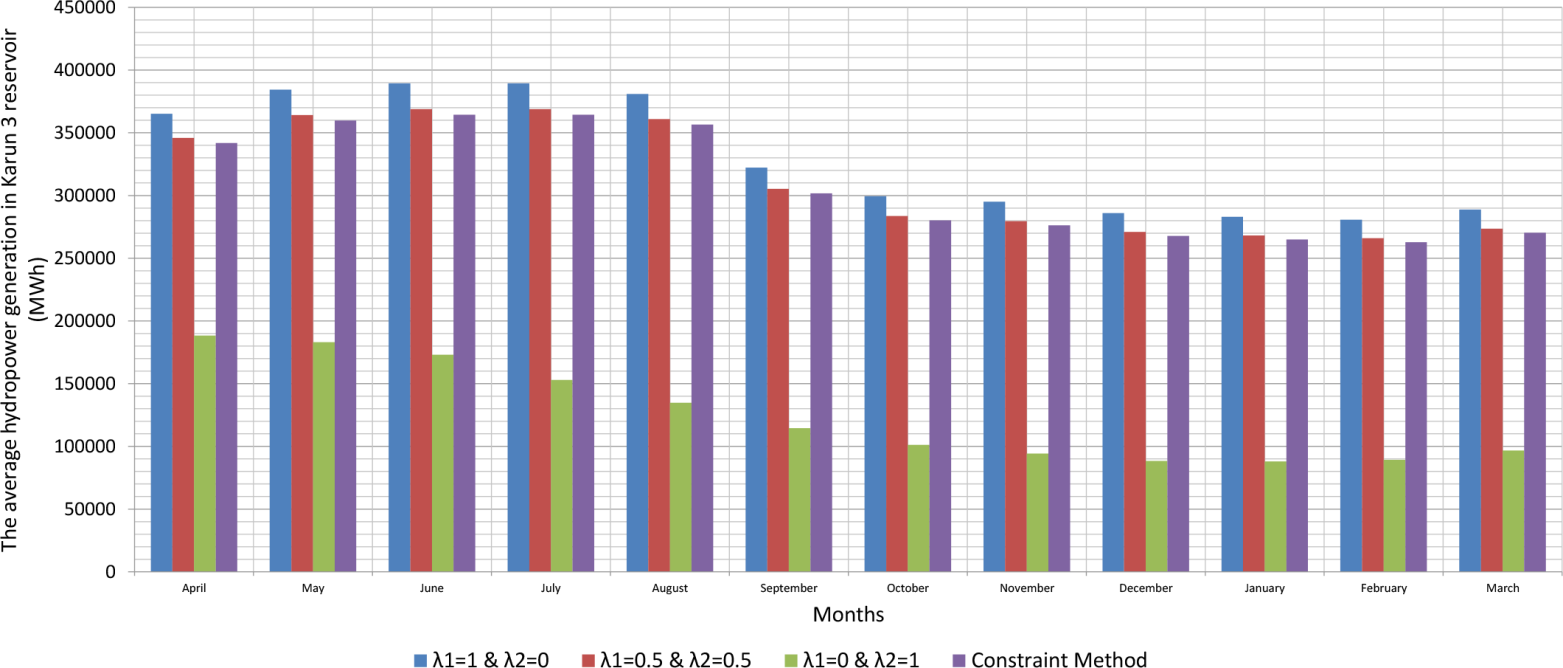
The average hydropower generation in Karun 3 reservoir (MWh).

**Fig 7:**
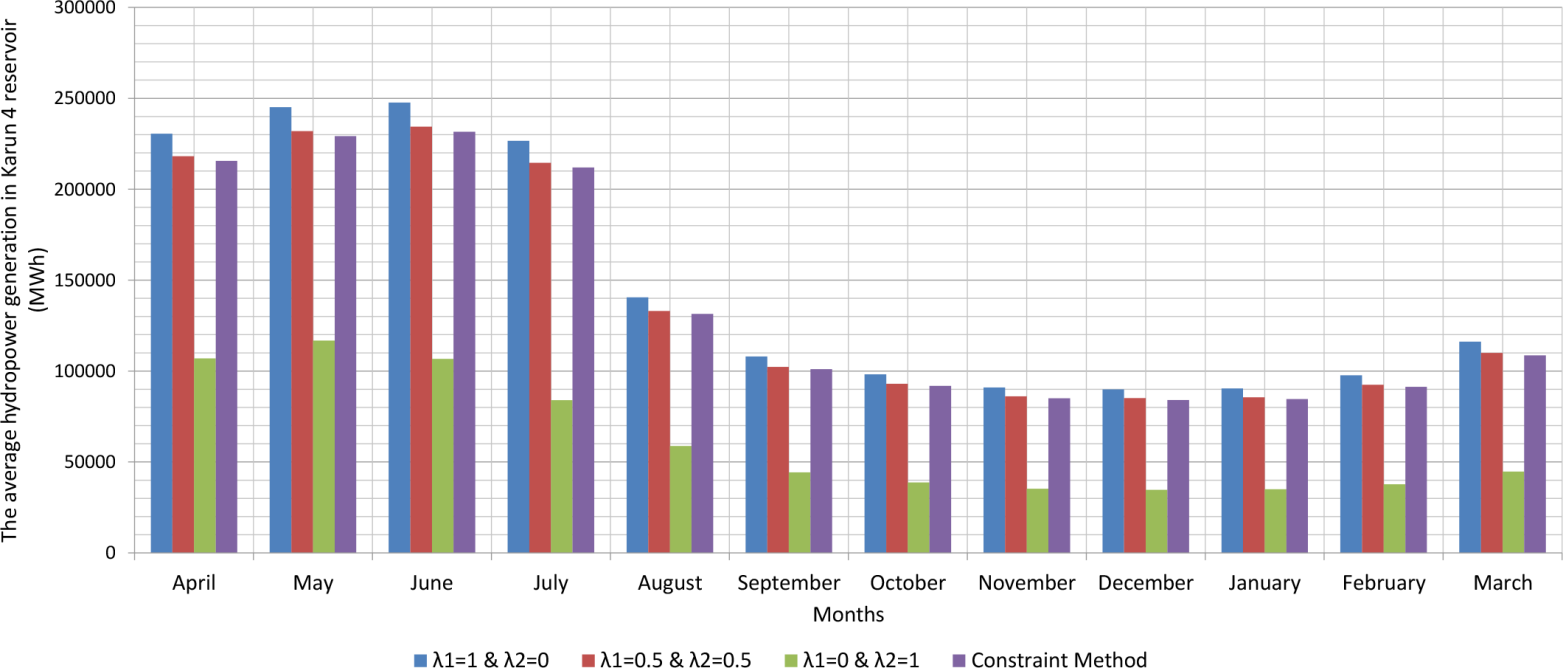
The average hydropower generation in Karun 4 reservoir (MWh).

Since, the production of hydroelectric energy is a function of the reservoir volume and release rate, the weighted objective function for energy production is one. The weighted objective function for agricultural and industrial needs is zero (Table 1). Therefore, large amounts of water stored behind the dam exit from the turbines. On the other hand, the increased volume of water stored behind the dam will increase the amount of energy produced per month. Hydroelectric power production is the function of the height of the water stored behind the dam and the volume of water passing through the turbine. However, increasing the water release rate for hydroelectric power production is relatively more effective than increasing the height of water stored behind the dam because of the physical structure of reservoir dams and location of turbines. When the volume of water stored behind the dam is close to its maximum and it is not possible to drain the water from the tunnel, there will be an overflow through sub-outputs. With the increased volume of water passing through the turbines, the water level increases in the tailwater. Increasing water height in the tailwater will decrease hydroelectric power production. For this reason, it is not so often possible to release the water to the extent of the water capacity of the tunnels. A comparison between the results of the optimization model and scenarios for implementing the method of Weighting is presented in the Figs 2, 3, 4, 5, 6 and 7.

**Table 1:** The average reservoir storage, release rate, overflow rate and proportion of water demand of Karun 1 (while: λ1 = 1 & λ2 = 0).

	Apr	May	Jun	Jul	Aug	Sep	Oct	Nov	Dec	Jan	Feb	Mar
**Storage (MCM)**	2470	2950	3100	2936	2727	2525	2337	2205	2160	2144	2056	2039
**Release (MCM)**	2000	2000	2000	1896	1407	1279	1244	1197	1103	1083	1156	1128
**Overflow (MCM)**	237	158	65	0	0	0	0	0	0	0	0	0
**Proportion of demand (%)**	38%	34%	42%	35%	27%	26%	29%	46%	56%	54%	46%	32%

The implementation of this scenario (Table 1) determined that the volume of seasonal flooding increases solely in the spring, and overflows through sub-outputs and the discharged water volume is very low through these sub-outputs in the other months. The percentage of consumption needs of an area is estimated at 35–40% which has the lowest value of 26% in September and the highest value of 56% in December. Hydroelectric energy is produced to the highest and lowest level, in the months of June and January with a production value of 327242 (MWh) and 160070 (MWh) energy, respectively.

By weight loss of objective function of hydroelectric energy production and weight gain of the objective function of consumer needs, it is possible to reduce the energy production which will increase the percentage of demand for water supplied (Tables 2, 3, 4 and 5). By examining the results of different scenarios, it can be determined that for weight changes of the objective functions, rate of changes in volume of stored water, the rate of release, the rate of overflow, the demand percentage of water supply and the rate of hydroelectric power production are negligible.

**Table 2:** The average reservoir storage, release rate, overflow rate and proportion of water demand of Karun 1 (while: λ1 = 0.5 & λ2 = 0.5).

	Apr	May	Jun	Jul	Aug	Sep	Oct	Nov	Dec	Jan	Feb	Mar
**Storage (MCM)**	2519	2974	3100	3079	2813	2466	2273	2184	2179	2199	2125	2039
**Release (MCM)**	2000	2000	2000	1554	1226	1234	1145	1150	1073	1056	1125	1131
**Overflow (MCM)**	45	52	38	0	0	0	0	0	0	0	0	0
**Proportion of demand (%)**	72%	71%	75%	70%	68%	68%	70%	76%	80%	%80	77%	72%

**Table 3:** The average reservoir storage, release rate, overflow rate and proportion of water demand of Karun 1 (while: λ1 = 0 & λ2 = 1).

	Apr	May	Jun	Jul	Aug	Sep	Oct	Nov	Dec	Jan	Feb	Mar
**Storage (MCM)**	1976	2376	2603	2541	2277	1979	1778	1832	1933	2049	2121	2104
**Release (MCM)**	633	642	565	508	487	457	409	331	304	299	311	353
**Overflow (MCM)**	2282	1816	1217	933	803	698	574	419	369	361	385	481
**Proportion of demand (%)**	94%	93%	94%	93%	94%	93%	94%	94%	95%	95%	95%	94%

**Table 4:** The average results of hydroelectric power generation of Karun 1 using weighting method (MWh).

λ1	λ 2	Apr	May	Jun	Jul	Aug	Sep	Oct	Nov	Dec	Jan	Feb	Mar
**1**	**0**	310644	324268	327242	302163	220171	216099	196492	177803	162030	160070	173324	177238
**1**	**0.5**	295704	308672	311504	287631	209582	205706	187042	169252	154237	152372	164988	168713
**0**	**1**	90854	95559	85849	76858	71993	65815	57903	47057	43677	43355	45440	51476

**Table 5:** The average results of hydroelectric power generation of Karun 1 using ε–constraint algorithm (MWh).

	Apr	May	Jun	Jul	Aug	Sep	Oct	Nov	Dec	Jan	Feb	Mar
**Supplying 80% of the water demand**	292162	304974	307772	284185	207071	203241	184801	167224	152390	150546	163012	166692

In a scenario where the weight of objective function for hydroelectric power production is equal to zero and the weight of function for the demand for water supplied is equal to one (Table 3), water stored behind the dam is discharged in order to meet the needs of areas of use. The rate of water discharge will be set in different months in such a way that it is always possible to meet the needs of areas of use in dry months. In these periods there is the possibility of overflow, due to seasonal flooding and increasing the volume of stored water, large amounts of water to the dam will overflow.

It is clear with the implementation of this scenario that the needs of agriculture and industries will increase dramatically and about 90 percent of consumer needs will be met. In this case, the average production of hydroelectric energy is about half of the generated energy resulted from the first scenario implementation per month (the scenario where weighted energy function is equal one and the weight of water demand function is equal zero). Maximum energy production takes place in May while the minimum energy is produced in November. Energy production is 95559 (MWh) in May and 43355 (MWh) in January (Table 4).

The general optimization modeling of Karun-Dez system has only two modes. If the objective function is only a function of demand for water supply, large amounts of water are stored behind the dam to meet consumer needs in the dry months. In the case that the model also has hydroelectric power production function, large amounts of water stored behind the dam is discharged. Finally, the survey results indicated that the structure of the objective function of hydroelectric energy production plays a more important role than the function of demand for water supply in providing the optimal solutions and non-low points.

### ε—constraint method algorithm

In this method, one of the objective functions is selected for optimization and other objective functions turn to the constraint with an upper boundary of epsilon.

$$\begin{array}{l} Min\;f_{i}\left(x\right)\\ S.t.\;f_{j}\left(x\right)\leq \;\varepsilon_{j}\;;\forall j\neq i \end{array}$$ (56)

To apply epsilon constraint method, we must specify the interval of objective functions f_j_(x) to initialize a value for ε_j_ in this interval. If the optimization problem has “i” objective functions, then (i-1) of objective functions should be considered as constraints. In this method, to find out more Pareto answers, εj should gradually be increased and solve the problem again.

In the optimization model of Karun-Dez dams, the objective function of demand for water supplied is entered as the limit into limit set and then the model is optimized by objective function of hydroelectric energy production. A set of non-low answers of the problems will be defined by changing low bounds of demand for water supply.

Model (50 and 51) is converted into a single-objective programming problem by using **ε**-constraint method.

$$MaxF_{1}={\sum_{i=1}^{6}\sum_{t=1}^{T}Power}_{i}(t)$$ (57)

$$s.t.:F_{d}\leq 0$$ (58)

$$\sum_{j=1}^{7}\sum_{t=1}^{T}\left(dem_{j}\left(t\right).\alpha_{j}\left(t\right)-dem_{j}\left(t\right)\right)\leq \sum_{j=1}^{7}\sum_{t=1}^{T}\left(dem_{j}\left(t\right).\alpha_{j}\left(t\right)-dem_{j}\left(t\right)\right)$$ (59)

In order to implement the **ε**—constraint method, the algorithm in the model (59), the range of changing the lower bound for the objective function of water supply was considered as follows:

$$0\leq \alpha_{j}(t)\leq 1$$ (60)

Firstly, two single-objective programming problems will be optimized by the implementation of the Ɛ- constraint method algorithm and the values for each of the objective functions was calculated based on the optimal value of the objective function. The length of step provided equaled k = 0.1 for changes in lower bounds and finally, the set of non-low responses were defined for the problem. The survey results indicated that if the lower bounds of the objective function of providing water demand is closer to the number 1, the flow through the turbine will be reduced and the volume of water stored behind the dam will be raised. If the bounds are also closer to the number 0, the flow passing through the turbine will be increased. By making smaller ranges of variation for the lower bounds and reducing the length of steps for these changes, it was determined that if 0.75≤ a_j_ (t) ≤0.90, in addition to increasing the amount of electrical energy production it is possible to meet a high percentage of agricultural and industrial needs in the area of consuming. A review of the obtained results of implementation of this scenario shows that in this case, the volume of stored water is very high as the obtained results of implementation of the scenarios of weighting method. On the other hand, in this scenario the rate of flow passing through the turbine is far less than the rate of release of the implementation of the method of weighting scenario’s. As a result, the amount of energy generated from the implementation of this scenario declines in comparison with the energy generated from the first scenario of the weighting method. Providing at only 80% of the agricultural and industrial needs is considered as a constraint of the model, therefore, in different months (even dry months and low rainfall months), an average 85% of the area of consumer needs to be met. The other results include the dramatic decrease in the average volume of water flowing in the months with high water level. Most of the energy produced in this case is done in June with a value of 307772 (MWh) and the lowest in January with a value of 150546 (MWh) (Table 5).

Figs 2, 3, 4, 5, 6, and 7 show a comparison between the results of the implementation of three scenarios of weighting methods and one scenario **ε**-constraint method for all reservoirs. As it can be seen, it is possible to increase the production of hydroelectric power and meet a high percentage of agriculture and industry needs by the implementation of the **ε**—constraint method algorithm.

## Conclusion

The study also plans to maximize hydroelectric power production and water supply for industrial and agricultural needs by using multi-criteria programming which has been modelled and optimized, regarding the operation of the set of Karun-Dez reservior dams which are the main system of reservior dams in Iran. Implementation of this model has been carried out by using sparse matrix structure. From evaluation of optimization time due to its high dimensions regarding the number of limitations and decision variables in a 40-year period, it was identified that implementation of the matrix structure and using interior point algorithms are appropriate tools to optimize linear and nonlinear models with large dimensions. Then, computational results were obtained by solving the model using different scenarios for combinations of the objective functions which were studied by using weighting methods algorithms and Ɛ- constraint method algorithms. After reviewing these results, it was found that the impact of hydroelectric power is quite impressive in the determination of the optimum solution in comparison with the function of providing water demand. These results provide a useful means to decide for the optimal amount of flow passing through the turbines, the rate of water flowing and the amount of water supply for agricultural and industrial purposes in the short term and long term planning for executives and decision-makers. Also, in the future research work, we can use multi-objective evolutionary optimization algorithms such as NSGA-II and MOPSO methods to resolve and compare the accuracy of the obtained results.

## Supporting Information

S1 Table Table of Result: https://figshare.com/s/99f0813e89ef16bdf6ba, DOI: 10.6084/m9.figshare.2069088. (XLSX)
